# Senolytics (DQ) Mitigates Radiation Ulcers by Removing Senescent Cells

**DOI:** 10.3389/fonc.2019.01576

**Published:** 2020-02-14

**Authors:** Huilan Wang, Ziwen Wang, Yu Huang, Yue Zhou, Xiaowu Sheng, Qingzhi Jiang, Yawei Wang, Peng Luo, Min Luo, Chunmeng Shi

**Affiliations:** ^1^Department of Oncology, The Affiliated Hospital of Southwest Medical University, Luzhou, China; ^2^State Key Laboratory of Trauma, Burns and Combined Injury, Institute of Rocket Force Medicine, Third Military Medical University, Chongqing, China; ^3^Department of Toxicology, Key Laboratory of Environmental Pollution Monitoring and Disease Control, Ministry of Education, Guizhou Medical University, Guiyang, China; ^4^Hunan Branch Center, National Tissue Engineering Center of China, Translational Medical Center, Central Laboratory, Hunan Cancer Hospital and The Affiliated Cancer Hospital of Xiangya School of Medicine, Central South University, Changsha, China

**Keywords:** radiation ulcer, oral mucositis, skin ulcer, senescence, apoptosis, SASP

## Abstract

Radiation ulcers are a prevalent toxic side effect in patients receiving radiation therapy. At present, there is still no effective treatment for the complication. Senescent cells accumulate after radiation exposure, which can induce cell and tissue dysfunction. Here we demonstrate increased expression of p16 (a senescence biomarker) in human radiation ulcers after radiotherapy and radiation-induced persistent cell senescence in animal ulcer models. Furthermore, senescent cells secreted the senescence-associated secretory phenotype (SASP) and induced cell senescence in adjacent cells, which was alleviated by JAK inhibition. In addition, the clearance of senescent cells following treatment with a senolytics cocktail, Dasatinib plus Quercetin (DQ), mitigated radiation ulcers. Finally, DQ induced tumor cell apoptosis and enhanced radiosensitivity in representative CAL-27 and MCF-7 cell lines. Our results demonstrate that cell senescence is involved in the development of radiation ulcers and that elimination of senescent cells might be a viable strategy for patients with this condition.

## Introduction

Radiation therapy is a common and efficacious treatment for patients with solid cancers. About 50% of cancer patients receive radiation therapy, alone or in combination with other treatment methods such as surgery (1). Among them, radiotherapy is the main treatment method for patients with head and neck tumors and has varying success (2), but oral mucositis is a crucial dose-limiting toxic effect (3). Radiotherapy is an important adjuvant treatment after surgery for breast cancer and can reduce the metastasis and mortality rates (4), but high-dose radiation exposure to superficial tissue ultimately leads to intractable skin ulcers. Although advances in radiotherapy such as dynamic intensity-modulated radiotherapy achieve precise delivery of radiation to cancer cells, side effects to surrounding tissues are still inevitable and bring great pain and/or cost to patients (5).

Various precautionary methods and therapies such as anti-inflammatory agents, local anesthetics, and growth factors have been used to treat painful ulcerations, but the clinical effects are poor (2). Palifermin, a recombinant human form of keratinocyte growth factor (KGF), is the only U.S. Food & Drug Administration–approved agent that is used to prevent oral mucositis in patients with bone marrow transplantation, but fibroblast growth factor receptor 2b (FGFR2b) is often overexpressed in cancer cells and increases the risk of tumor growth (6). Although hyperbaric oxygen therapy has been reported to reduce skin ulcers after radiation (7), the treatment duration is long. Therefore, the development of potential agents that mitigate radiation ulcers without accelerating tumor growth is intensively needed for oncological supportive care.

Cell senescence can be triggered by radiation-induced DNA damage and leads to delayed repair and regeneration of irradiated tissue (8). Persistent damage activates the cyclin-dependent kinase inhibitor p16^Ink4a^ and causes cell cycle arrest (9). Cellular senescence is not just a state of proliferation inhibition and genetic alteration (10); senescent cells can secrete cytokines, called the senescence-associated secretory phenotype (SASP) including inflammatory factors (11, 12), tissue-reconstituted proteases, and growth factors, which can induce persistent chronic inflammation in the tissue microenvironment (13, 14) and promote cancer relapse (15). It has been reported that cordycepin and mammalian target of rapamycin inhibition can protect from radiation ulcers by inhibiting cell senescence (16, 17). These observations led us to explore if it is possible to mitigate radiation ulcers by eliminating senescent cells.

In this study, we show that senescent cells persist in radiation ulcers (clinical radiation ulcer samples and animal ulcer models), and clearance of senescent cells by the senolytics drug cocktail, dasatinib plus quercetin (DQ), can effectively mitigate radiation ulcers. Moreover, DQ treatment can enhance cancer cell radiosensitivity. Our findings suggest that cell senescence is involved in radiation ulcer development, and clearance of senescent cells can be a potential therapeutic method to mitigate radiation ulcers.

## Materials and Methods

### Human Skin Samples

Skin tissues were obtained from healthy volunteers and patients with breast cancer receiving radiation therapy from 2016 to 2018 at Hunan Cancer Hospital (the Affiliated Hospital of Xiangya School of Medicine of Central South University). Skin ulcer samples were obtained from the chest wall at the time of surgery and were processed for further analysis. The studies involving human participants were approved by the ethics committee of Hunan Cancer Hospital; the patients/participants provided their written informed consent to participate in our study.

### Cell Culture

Human oral keratinocytes (HOK ATCC, PCS-200-014) were cultured in an oral keratinocyte medium containing antibiotics at 37°C in 5% CO_2_. Human fibroblasts, CAL27 (CRL-2095), and MCF-7 cells (ATCC, HTB-22) were cultured in Dulbecco's minimum essential medium with high sugar (Invitrogen) supplemented with 10% fetal bovine serum (Gibco) and 1% streptomycin/penicillin. The isolation protocol for human fibroblasts was described previously (18).

### Conditioned Medium (CM)

CM was made by exposing young cells to a fresh medium for 24 h. SASP-CM was made by exposing senescent cells (7 days after radiation) to a fresh medium for 24 h. To collect (SASP+JAKi)-CM, senescent cells were treated with JAK inhibitor 1 (JAKi) or dimethyl sulfoxide (DMSO) for 72 h and cultured with a fresh medium containing JAKi or DMSO for another 24 h.

### Animal Models

Female C3H mice (6–8 weeks) and male Sprague–Dawley rats (6–8 weeks) were purchased from Laboratory Animal Center of Army Medical University. To evaluate the effect of senolytics on radiation ulcers, animals were divided into non-radiation, radiation, and D+Q treatment groups. For local fractionated radiation, the head and neck area was exposed to irradiation at a dose of 6 Gy/day (X-RAD 160-225 instrument Precision X-Ray, 1.9 Gy/min) and treated with senolytics dasatinib (5 mg/kg) plus quercetin (50 mg/kg) (D+Q) (19, 20) by oral gavage every day for 5 days. Mice were sacrificed at days 3, 6, 8, and 10. For skin ulcer modeling, rats' right posterior limbs were exposed to a single dose of 40 Gy (0.9 Gy/min) radiation and treated with dasatinib (5 mg/kg) plus quercetin (50 mg/kg) (D+Q) by intraperitoneal injection every day for 5 days after irradiation. Rats were sacrificed at days 5, 8, 11, and 15 after irradiation.

### Immunoanalysis and Histopathology

Tissues were fixed, embedded in paraffin, cut into 3-μm sections, and stained with hematoxylin and eosin (H&E). For p16 immunohistochemistry, slides were boiled in a citrate buffer for antigen retrieval after dehydration. Slides were then soaked in 10% hydrogen peroxide for 10 min to remove endogenous peroxidase and were washed. Slides were blocked in goat serum and incubated in primary antibody against p16 (Abcam, 1:100) at 4°C overnight. Washed slides were then incubated with secondary antibody for 40 min (biotinylated goat anti-rabbit IgG, BA-1000, Vector Labs), washed, and incubated in 3'-diaminobenzidine solution. For γ-H2AX and Ki67 immunofluorescence, antigen retrieval and blocking was performed as above, and primary antibody (γ-H2AX, Cell Signaling, 1:200; Ki67, Cell Signaling, 1:200) was applied and incubated at 4°C overnight. Slides were washed with phosphate-buffered saline and incubated with secondary antibody for 40 min (biotinylated goat antirabbit IgG, 594 nm) before adding an antifluorescence buffer containing 4′,6-diamidino-2-phenylindole for imaging.

### Real-Time qPCR

Total RNA from tissues or cells was extracted using TRIzol (Life Technologies) and reverse-transcribed to cDNA using the Maxima First Strand cDNA Synthesis Kit (Thermo Scientific, K1671). Real-time PCR was performed by applying the SYBR Green (Takara) qPCR master mix following the manufacturer's protocol. ΔCt values were calculated as the following formula: ΔCt = Ct target – Ct actin. Values of sample reference to control were calculated using the ΔΔCT method; the difference of gene expression was calculated using the 2^−(ΔΔCt)^ formula. qRT-PCR primer sequences are shown in Supplementary Table 1. Actin was used as an internal control.

### SA-β-Gal Activity

Cells were seeded into 6-well plates and then either received 8-Gy (0.9 Gy/min) radiation or not. Cells were passed and assessed 7 days after radiation. SA-β-gal staining was done using a SA-β-gal staining kit (Cell Signaling) according to the manufacturer's instructions. First, 1 ml 4% paraformaldehyde was added to every plate to fix cells. Then, cells were incubated at 37°C for 24 h in a SA-β-gal staining solution (pH = 6.0, Cell Signaling). Blue-stained cells were senescent cells.

### Flow Cytometry

Cells were seeded into 6-well plates at a density of 2 × 10^5^ cells/well. Cells either exposed to radiation (8 Gy) or not were treated with DMSO or DQ (1 mM D+20 mM Q) for 24 h, digested with trypsin, and collected. Cells were then resuspended in a 100-μl binding buffer with 1-μl fluorescein isothiocyanate Annexin-V and 1-μl propidium iodine (PI; BD Biosciences, 556547). Finally, samples were analyzed by flow cytometry (C6, BD Biosciences, San Jose, CA). For cell cycle analysis, cells were fixed with Fixation/Permeabilization Diluent/Concentrate (eBioscience) for 30 min. Subsequently, intracellular Ki-67 (eBioscience) and Hoechst33342 (Sigma) staining were performed using PermWash solution (eBioscience). Cells were washed once prior to flow cytometry analysis.

### Western Blot

Cells were extracted in a cell lysis buffer (Cell Signaling) with protease inhibitors (Sigma). Proteins were loaded into each lane on a 5–12% gradient sodium dodecyl sulfate/polyacrylamide gel and transferred to immunoblot polyvinylidene fluoride membranes (Bio-Rad). Membranes were blocked with 5% skim milk and probed with primary antibodies at 4°C overnight. Horseradish peroxidase-conjugated secondary antibodies (Beyotime) were applied for 1 h at room temperature. The band intensities were visualized and quantified using an enhanced chemiluminescence detection system (Bio-Rad Laboratories). Primary antibodies used were as follows: poly ADP-ribose polymerase (PARP, 1:1,000, abcam), caspase 3 (1:1,000, abcam), cleaved caspase 3 (1:1,000, abcam), p-JAK1(1:1,000, abcam), p-JAK2 (1:1,000, abcam), and β-actin (1:1,000, Beyotime).

### Enzyme-Linked Immunosorbent Assay (ELISA)

The concentrations of human inflammatory cytokines from HOK and fibroblasts cell supernatant were measured with ELISA kits. IL-1α (KE00123), IL-6 (KE00139), IL-1β (KE00021), IL-8 (KE00006), and tumor necrosis factor (TNF)-α (KE00154) ELISA kits from ProteinTech were used following the manufacturer's protocols. Generating a linear standard curve based on the OD value of the standard, the expression of protein was calculated using the formula generated above.

### Statistical Analysis

Comparisons between two groups were analyzed using unpaired Student's *t*-tests, and values are presented as mean with SD. Statistical significance was set as ^*^P < 0.05, ^**^P < 0.01, and ^***^P < 0.001. SPSS 13.0 statistical software was used to perform all statistical analyses, and GraphPad Prism 7.0 was used to generate graphs.

## Results

### Senescence Biomarkers Accumulate in Human Radiation Ulcer After Radiotherapy

**Figure 1:**
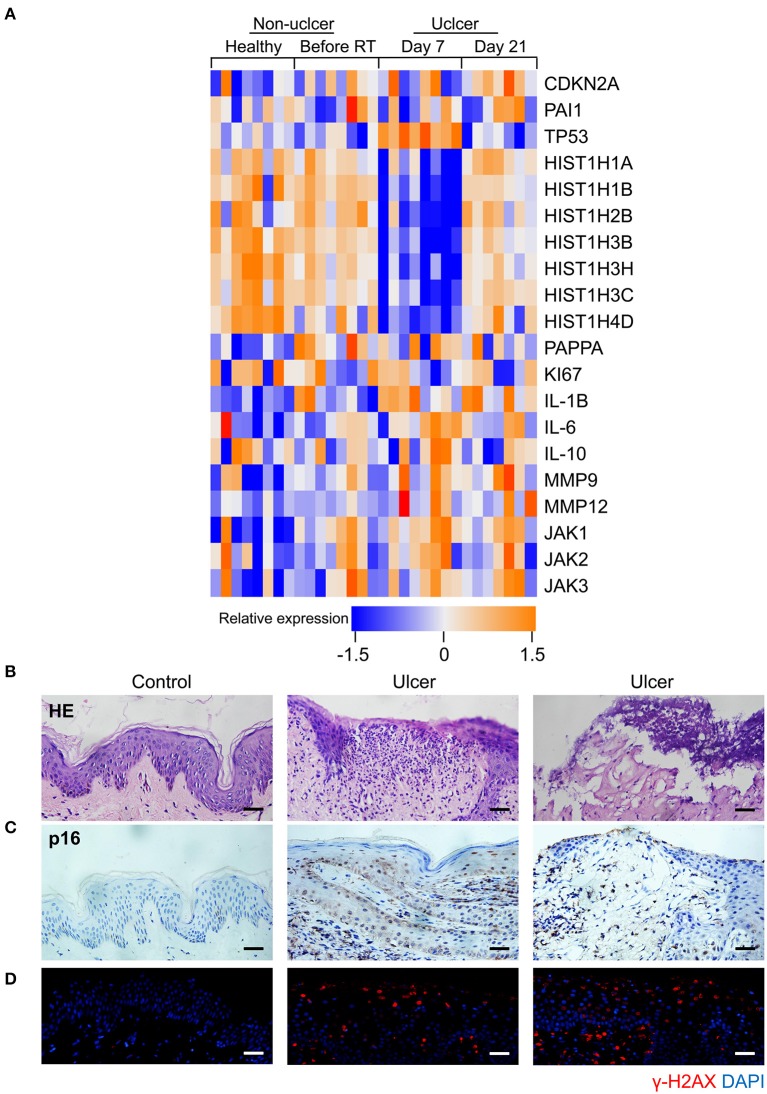
Senescence biomarkers accumulate in human radiation ulcer after radiotherapy. **(A)** Heat map showed the expression of senescence, DNA damage, and SASP genes in mucositis in patients with tonsil squamous cell carcinoma (during and after radiation therapy) and control (healthy mucosa and patient before radiotherapy) human cohorts (healthy *n* = 8, before radiation *n* = 8, day 7 *n* = 8, day 21 *n* = 7). **(B)** Histological analysis of skin tissues from healthy volunteers and radiotherapy patients. **(C)** Immunohistochemistry staining of p16 of skin tissues from healthy volunteer and radiotherapy patients. **(D)** Immunofluorescence staining of γ-H2AX of skin tissues from healthy volunteer and radiotherapy patients. **(B–D)** Healthy *n* = 1, radiotherapy patients *n* = 4, skin tissue from the chest wall; scale bar, 50 μm.

Senescence can be induced by multiple mechanisms such as DNA damage, reactive oxygen species (ROS) production, and oxidative stress (21), and DNA damage is a critical mediator of cellular alterations caused by radiation exposure (22). To explore the hypothesis that cell senescence and SASP are related to human radiation ulcers after radiotherapy, we first analyzed established senescence genes in the GSE103412 dataset (23) corresponding to mucositis in patients with tonsil squamous cell carcinoma (during and after radiation therapy) and control human cohorts (healthy mucosa and patients before radiotherapy). CDKN2A (p16) and TP53 were upregulated within oral mucosa samples of individuals with mucositis during and after radiation therapy (Figure 1A). In addition, HIST1H3B, HIST1H2BM, HIST1H3C, HIST1H3H, HIST1H1A, HIST1H4D, and HIST1H1B were downregulated (Figure 1A) in mucositis samples, especially at day 7 after radiation. This is notable since histone gene expression downregulation is a response to DNA damage (24). Ki67 (a marker of proliferation) was downregulated, indicating that radiation decreased the proliferative capacity of mucosa. Based on the hypothesis that senescent cells promote the development of radiation ulcers through the secretome, we analyzed the expression of SASP genes in human mucositis transcriptome datasets (GSE103412). Expression of pregnancy-associated plasma protein A (23), several matrix metalloproteinases (MMPs), and interleukin (IL) family members were also increased after radiation therapy (Figure 1A).

We also immunohistochemically detected p16 and γ-H2AX in skin tissue samples from healthy volunteers and patients with breast cancer receiving radiation therapy. As shown in Figure 1B, a lack of epithelium in the tissue was observed in ulcer tissue samples compared to normal skin. We also found a remarkable increase in the senescence marker p16 (Figure 1C) and the DNA damage marker γ-H2AX (Figure 1D). Collectively, our results indicate that senescence biomarkers accumulate in human radiation ulcers after radiotherapy, and senescence may play a critical role in promoting human radiation ulcers.

### Radiation Induces Persistent Cell Senescence in Animal Ulcer Models

**Figure 2:**
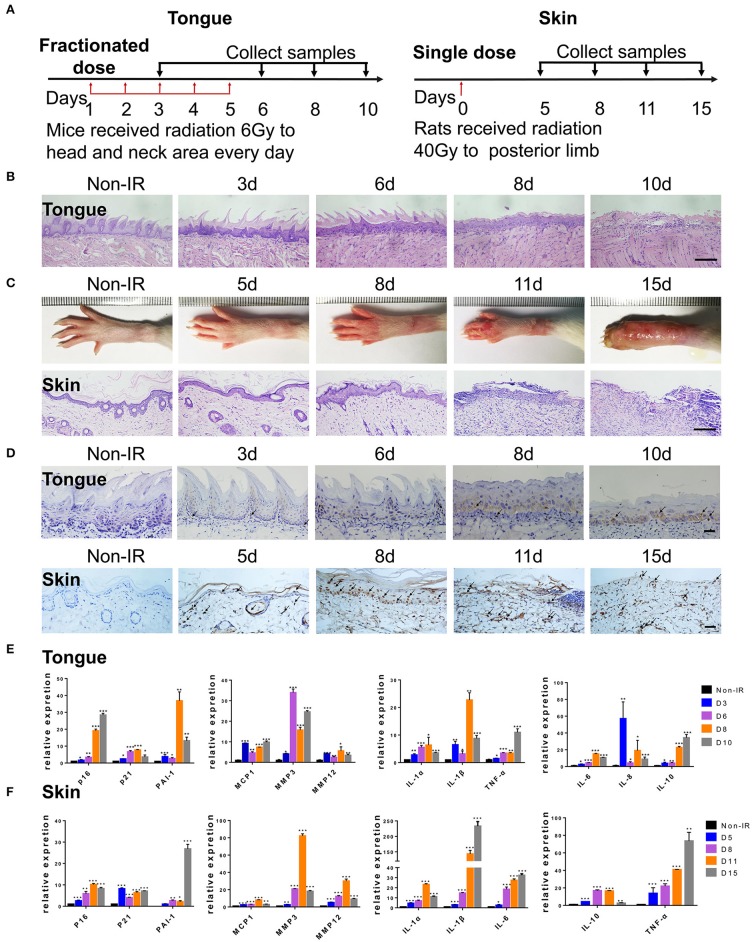
Radiation induces persistent cell senescence in animal ulcer models. **(A)** Radiation and drug treatment scheme for mice (left) and rats (right). **(B)** Histological analysis of mouse tongue tissues 0–10 days postradiation (*n* = 3). **(C)** Representative images of hind limb 0–15 days postradiation (top); histological analysis of rat skin tissues 0–15 days postradiation (bottom) (*n* = 3). **(D)** Immunohistochemistry of p16 in mouse tongue and rat skin tissues (*n* = 3). As indicated by the arrow, brown represents positive cells. **(E,F)** The expressions of p16, p21, PAI-1, and SASP genes (IL-1α, IL-10, IL-1β, TNF-α, IL-6, MMP3, IL-8, MMP12, and MCP1) in different time points were quantified by qRT-PCR (mean with SD; *n* = 3, *P < 0.05, **P < 0.01, ***P < 0.001; Student's *t*-test). **(B,D)** Scale bar, 100 μm; **(C)** scale bar, 50 μm.

To further confirm the correlation between radiation ulcers and cell senescence, a mouse oral ulcer and rat skin ulcer model were established (Figure 2A). For radiation-induced oral ulcers, the head and neck of mice were treated with fractionated radiation of a 6-Gy dose/day for 5 days (other body parts were covered with a lead board). Mice were euthanized at days 3, 6, 8, and 10, and the tongues were removed and analyzed. For radiation-induced skin ulcer, each rat's right posterior limb was exposed to a single 40-Gy radiation under anesthesia (25). As shown in Figures 2B,C, the irradiated tongues and skin exhibited severe destruction of the epithelial layer compared to normal epithelial morphology. Furthermore, both models showed increased immunohistochemical staining for the senescence marker p16 at different time points (Figure 2D). qRT-PCR showed that senescence markers p16, p21, and plasminogen activator inhibitor-1 (PAI-1) were increased in irradiated mice/rats (Figures 2E,F). We found that the SASP factors (26) [IL-1β, IL-6, IL-1α, IL-8, IL-10, TNF-α, MMP3, MMP12, and monocyte chemoattractant protein-1 (MCP1)] were all significantly upregulated in irradiated tongue and skin tissues compared to non-irradiated controls (Figures 2E,F). These results indicate that senescent cells and the SASP persist in radiation ulcer. These results are consistent with previously reported data for senescence-associated beta-galactosidase (SA-β-gal), a known marker of senescent cells (16). Therefore, eliminating senescent cells might be a viable strategy to alleviate radiation ulcers.

### Senescent Cells Induce Cell Senescence and SASP in Adjacent Cells

**Figure 3:**
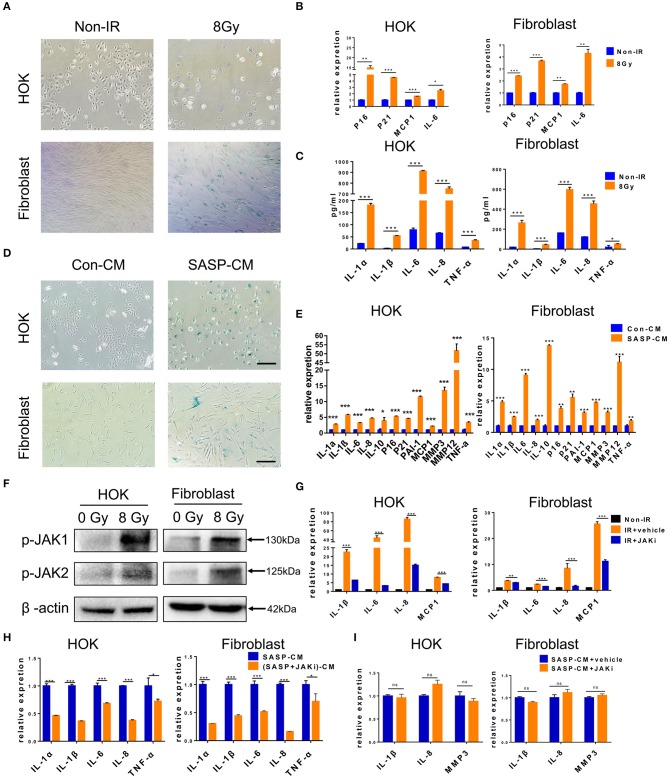
Senescent cells induce cell senescence and SASP in adjacent cells. **(A)** SA-β-gal staining in HOK and skin fibroblasts at 7 days after radiation (*n* = 3). **(B)** mRNA expression levels for p16, p21, MCP1, and IL-6 in HOK and skin fibroblasts at 7 days after radiation (*n* = 3). **(C)** Protein expression levels for IL-1α, IL-8, IL-6, IL-1β, and TNF-α in HOK and skin fibroblast cell supernatant (*n* = 3). **(D)** HOK and skin fibroblasts were cultured in Con-CM and SASP-CM for 7 days and assessed by SA-β-gal staining (*n* = 3). **(E)** mRNA expression levels of p16, p21, PAI-1, and SASP genes (IL-1α, IL-10, IL-1β, TNF-α, IL-6, MMP3, IL-8, MMP12, and MCP1) in HOK and skin fibroblasts (cultured in Con-CM and SASP-CM for 7 days). **(F)** p-JAK1 and p-JAK2 expression levels in HOK and fibroblasts after radiation. Three independent experiments started with cell plating. **(G)** Irradiation-induced senescent HOK and fibroblast were treated with JAK inhibitor and vehicle for 72 h. Then RNA was collected, and qRT-PCR was performed (*n* = 3). **(H)** Young HOK and skin fibroblasts were treated with SASP-CM and (SASP+JAKi)-CM for 24 h, respectively; the mRNA levels of SASP were analyzed. **(I)** mRNA levels of SASP in young HOK and skin fibroblasts, which were treated with SASP-CM, followed by addition of JAK inhibitor 1 or vehicle for 24 h. **(E)** IR+vehicle group compared with IR+JAKi group. **(E,G–I)** Mean with SD. *n* = 3, *P < 0.05, **P < 0.01, ***P < 0.001; Student's *t*-test. **(A,D)** Scale bar, 100 μm.

Senescent cells acquire autocrine/paracrine abilities, and the cytokines they produce promote dysfunction and growth arrest in neighboring cells to maintain senescence by an autocrine positive-feedback loop (27). Next, we tested whether senescent HOK and human skin fibroblasts induce senescence and inflammation in adjacent healthy cells. We first established an *in vitro* HOK and skin fibroblast cell senescence model induced by radiation (Figures 3A,B), which were confirmed by SA-β-gal staining (28) and the expression of senescence mediators (p21 and p16) and SASP factors (MCP1 and IL-6) (29). Morphologically, senescent HOK are larger and rounder, with more vacuoles and fewer antennae compared with young HOK. Young fibroblasts are spindle-shaped or polygonal, whereas senescent cells become larger, flat, and overstretched, with elongated branches at the ends of extensions (Figure 3A). Notably, IL-1α, IL-8, IL-6, IL-1β, and TNF-α protein expression levels were increased in cell supernatant from irradiated cells compared with non-irradiated cells (Figure 3C). Then, CM from senescent cell supernatant (SASP-CM) and normal cell supernatant (Con-CM) were collected; we found that exposure of non-senescent HOK and skin fibroblasts to SASP-CM for 7 days induced SA-β-gal expression and senescent morphology compared with Con-CM (Figure 3D). Cells were also collected for qRT-PCR analysis, which showed that CM derived from senescent cells caused upregulation of senescence genes (p16, p21, PAI-1) and SASP genes (IL-1α, IL-10, IL-1β, TNF-α, IL-6, MMP3, IL-8, MMP12, and MCP1) relative to CM from non-senescent cells (Figure 3E). These results indicate that the SASP can induce cell senescence and inflammation in adjacent cells.

The JAK pathway is important in cytokine production, and JAK1 and 2 primarily regulate inflammatory signaling (30). The GSE103412 dataset showed increased JAK1/2 in ulcer patients after radiation therapy (Figure 1A). Similarly, we found significantly increased JAK expression after irradiating HOK and skin fibroblasts (Figure 3F). We then assessed the effect of JAKi, which can suppress SASP in senescent cells by inhibiting the JAK pathway. Senescent HOK and skin fibroblasts were incubated with vehicle and JAKi (1 μM) for 72 h; then CM from senescent cells (SASP-CM) and senescent cells incubated with JAKi [(SASP+JAKi)-CM] were collected. The results showed that JAKi (1 μM) downregulated the expression of crucial SASP genes in senescent cells (Figure 3G). Furthermore, after young HOK and skin fibroblasts were treated with SASP-CM and (SASP+JAKi)-CM for 24 h, respectively, SASP mRNA levels were lower in the (SASP+JAKi)-CM group relative to SASP-CM-treated young cells (Figure 3H). Therefore, SASP in senescent cells may promote SASP in adjacent cells. However, when young HOK and skin fibroblasts were treated with SASP-CM, followed by the addition of JAKi or vehicle for 24 h, we did not observe decreased levels of SASP (Figure 3I). Therefore, we hypothesize that JAKi mainly acts on senescent cells by suppressing the SASP to reduce inflammation, but it has no effect on non-senescent cells to prevent inflammation caused by SASP. These findings demonstrate that senescent cells can induce cell senescence and SASP in adjacent cells, and JAK inhibition alleviates SASP in senescent cells.

### DQ Treatment Eliminates Senescent Cells by Inducing Apoptosis

**Figure 4:**
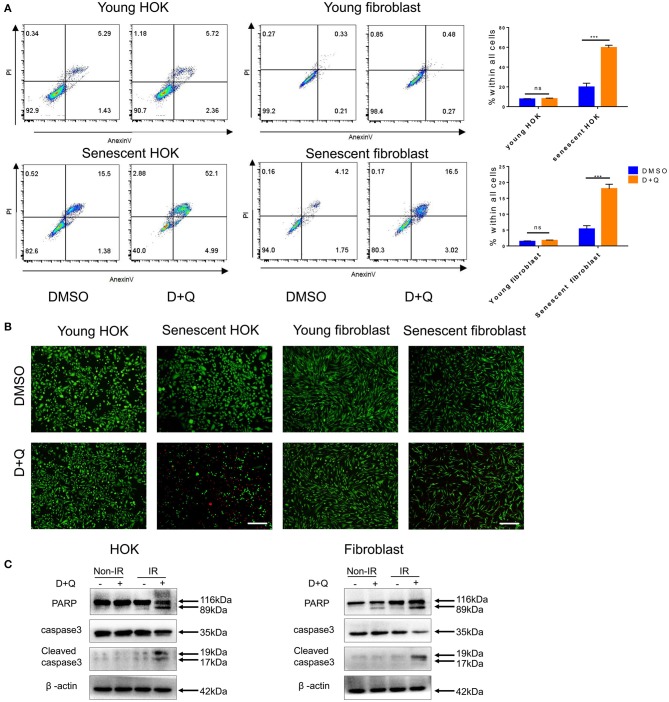
Senescent cells are eliminated by DQ treatment. **(A)** Young/senescent HOK and skin fibroblasts were treated with DMSO or DQ for 24 h, and collected for apoptosis analysis using flow cytometry (*n* = 3), repeated three times independently (mean with SD. *n* = 3, ***P < 0.001; independent samples Student's *t*-test; ns, no significance). **(B)** HOK and skin fibroblasts were co-stained with calcein-AM (Invitrogen)/PI to visualize live cells (green fluorescence) and dead or late apoptotic cells (red fluorescence) (*n* = 3; scale bar, 100 μm). **(C)** Apoptosis markers PARP, caspase3, and cleaved caspase3 expression levels in young/senescent HOK and skin fibroblasts after being incubated with DMSO or DQ for 24 h. Three independent experiments started with cell plating.

The above observations suggest that senescent cells may be a viable target in preventing radiation ulcers. Therefore, we assessed the effect of DQ, which has been reported to selectively clear senescent cells (11, 19, 20). We found that a single dose of DQ (1 mM dasatinib+20 mM quercetin) eliminated 40–60% of senescent HOK and 10–20% skin fibroblasts within 24 h; nevertheless, DQ treatment had no observable effect on young HOK or skin fibroblasts (Figure 4A). Similarly, calcein AM/PI staining showed markedly higher cell death in senescent HOK and fibroblasts compared to young cells (Figure 4B). Moreover, DQ induced the expression of the apoptosis markers caspase 3, cleaved caspase 3, and PARP in senescent cells (Figure 4C). These results suggest that DQ selectively removed senescent cells through the intrinsic apoptotic pathway.

### Senescent Cell Clearance Mitigates Radiation Ulcers

**Figure 5:**
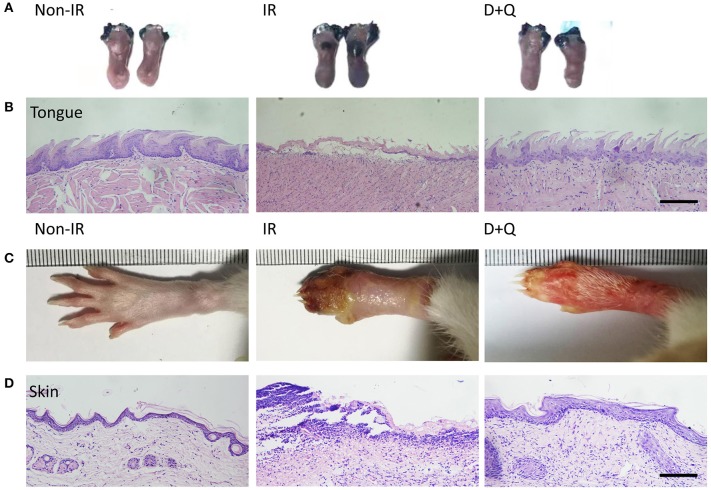
Senescent cell clearance mitigates radiation ulcer. **(A)** Toluidine blue staining pictures of mouse tongues at day 10 from non-radiation, radiation, and DQ treatment mice. Lack of integrated epithelial barrier (ulcer) presents blue staining (*n* = 5). **(B)** Histological analysis of mouse tongues from non-radiation, radiation, and DQ treatment mice (*n* = 5). **(C)** Images of posterior limbs from SD rats (non-radiation, radiation, and DQ treatment mice) on day 15 (*n* = 5). **(D)** Histological analysis of skin tissues from non-radiation, radiation, and D+Q treatment rats at day 15 (*n* = 5). **(B,D)** Scale bar, 50 μm.

**Figure 6:**
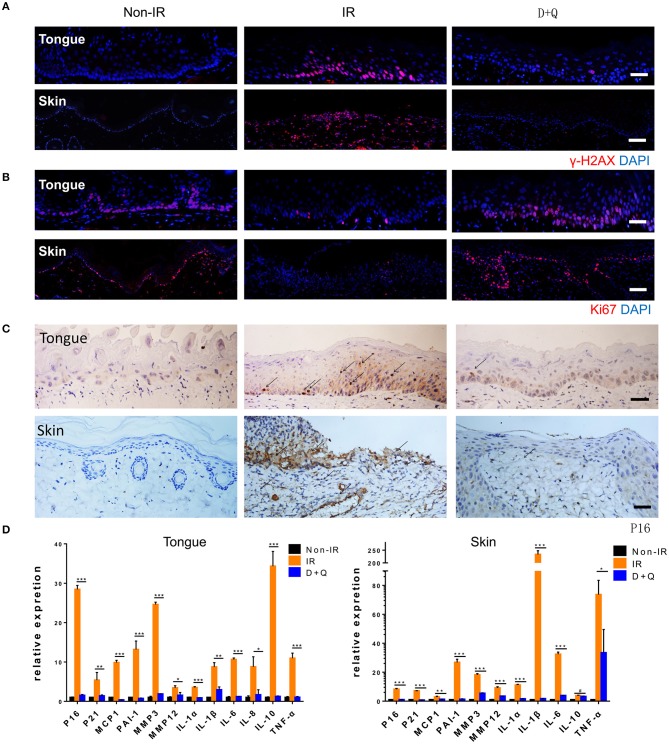
Senescent cell clearance mitigates radiation ulcer. **(A,B)** Immunofluorescence staining of γ-H2AX and Ki67 in mouse tongue and rat skin tissues from non-radiation, radiation, and D+Q treatment groups (*n* = 5). **(C)** Immunohistochemistry staining of p16 in mouse tongue and rat skin tissue (*n* = 5). **(D)** Quantification of mRNA expression for p16, p21, and SASP in mouse tongue tissues and rat skin tissues. **(D)** Mean with SD. *n* = 3, *P < 0.05, **P < 0.01, ***P < 0.001, # means no significance; Student's *t*-test. **(A–C)** Scale bar, 25 μm.

Next, we determined whether DQ could help heal radiation ulcers. DQ almost entirely prevented the appearance of mucositis in irradiated mice (Figure 5A). Histological analysis of the tongues showed complete and continuous epithelial layers in irradiated DQ-treated mice (Figure 5B). DQ also significantly decreased radiation-induced skin ulcers, desquamation, and edema and promoted epithelium repair (Figures 5C,D). In addition, we found reduced levels of the DNA damage response marker γ-H2AX in irradiated DQ-treated mice/rats (Figure 6A). Furthermore, DQ-treated mice/rats showed significantly increased levels of the proliferation marker Ki67 (31) (Figure 6B). As expected, DQ-treated mice/rats showed downregulation of the senescence marker p16 and SASP (Figures 6C,D). H&E staining showed that the heart, spleen, muscle, lung, intestine, kidney, and liver were not obviously affected by DQ treatment (Supplementary Figure 1), and there was no statistical difference in body weight between the DQ- and vehicle-treated groups after radiation (Supplementary Figure 2). These findings suggest the possibility that DQ treatment may alleviate DNA damage and maintain the proliferative capacity of tissue cells by eliminating senescent cells, thereby preventing the development of radiation ulcers.

### DQ Enhances Cancer Cell Radiosensitivity

**Figure 7:**
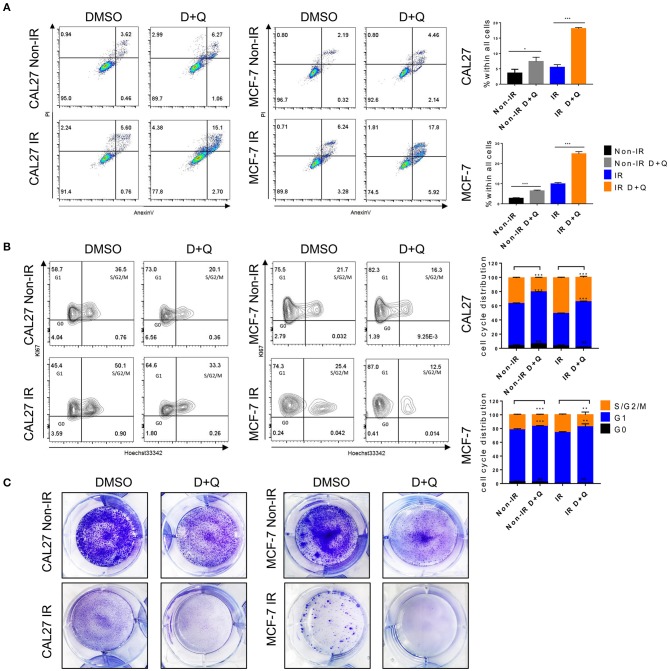
DQ enhances radiosensitivity of cancer cells. **(A)** Irradiated or non-irradiated CAL27 and MCF-7 were incubated with DMSO or DQ for 24 h, then cells were collected for analysis of apoptosis using flow cytometry. **(B)** Irradiated or non-irradiated CAL27 and MCF-7 cells were incubated with DMSO or DQ for 24 h; cell cycle was analyzed by flow cytometry. **(C)** Representative colonies of irradiated or non-irradiated CAL27 and MCF-7. **(A–C)** *n* = 3; repeated three times independently (mean with SD. *n* = 3, *P < 0.05, **P < 0.01, ***P < 0.001; Student's *t*-test).

Senescence induced by ionizing radiation can contribute to tumor therapy via cell growth arrest (32) and autophagy (33). It can antagonize apoptosis and consequently shelter a population of dormant cells, and this anti-apoptotic effect ultimately leads to cancer radiotherapy resistance (34) and tumor recurrence (35). In our study, we assumed that senescent cells including senescent tumor cells (irradiated tumor cells) might be viable targets of DQ. CAL27 and MCF-7 cells are used as typical examples of head and neck squamous cell carcinomas and breast cancer, respectively. CAL27 and MCF-7 cells were exposed to 8-Gy irradiation and then incubated with DQ for 24 h. Flow cytometry result showed that a single dose of 1 mM D+20 mM Q induced apoptosis of CAL27 and MCF-7 and promoted radiosensitivity (Figure 7A). We next assessed cell-cycle percentages using flow cytometry and found that cells in the G1 phase were significantly increased in non-irradiated DQ-treated CAL27 and MCF-7 cells compared with the control group treated with DMSO (Figure 7B). This phenomenon was also evident in irradiated cells (Figure 7B), indicating that DQ treatment induces cell-cycle arrest at G1 and S/G2/M checkpoints in CAL27 and MCF-7 cells. Proliferation was measured by colony formation assays, which showed that DQ reduced the colony formation ability of both CAL27 and MCF-7 cells (Figure 7C). Our results suggest that DQ induced tumor cell apoptosis and also enhanced radiosensitivity and reduced proliferative capacity in CAL27 and MCF-7 cells.

## Discussion

Radiation therapy is an indispensable treatment for tumors that is applied to approximately half of cancer patients with different effects. It achieves good results in the treatment of head and neck and breast cancers. The radiation dose is determined by the sensitivity of the tumor and surrounding tissues (36). Oral mucositis is a crucial dose-limiting toxic effect in radiotherapy for head and neck cancers (37), and skin ulcers are a common side effect in patients with breast cancer (38, 39). Radiotherapy induces DNA strand breaks, ROS production, and oxidative stress that eventually trigger cell senescence and amplify acute damage (9, 40, 41). Our results show that senescence biomarkers accumulate in human radiation ulcers after radiotherapy. Moreover, the expression of senescence-related genes and proteins was significantly increased after radiation and accumulated over time in radiation-induced ulcer models.

Cellular senescence is a cell-intrinsic program, and there is considerable evidence that senescent cells can affect neighboring cells and surrounding environment via their SASP (42, 43). In this study, senescent cells induced senescence and the SASP in adjacent cells, and JAK inhibition alleviated the SASP in senescent cells. Therefore, we reasoned that senescent cells may be a viable target in alleviating radiation ulcer. Furthermore, we found that DQ mitigated radiation ulcers via the removal of senescent cells. We previously reported that cordycepin prevented radiation ulcers by inhibiting cell senescence, and in this study, we showed that removal of senescent cells by DQ effectively ameliorated radiation ulcers. Therefore, inhibiting cell senescence or clearing senescent cells can be a therapeutic strategy in mitigating radiation-induced ulcers. Plausibly, JAK inhibition can also be used to treat irradiation ulcers by alleviating the SASP; however, JAK inhibition needs to be continuously administered daily to maintain SASP inhibition. For this purpose, DQ would be administered several times (e.g., once monthly) to minimize senescent cells (19). Importantly, JAK inhibition causes severe side effects after discontinuation, including cardiogenic shock, tumor lysis syndrome, and even life-threatening events, but there are no obvious side effects after DQ treatment discontinuation (11, 44). Hence, DQ treatment is a better choice for mitigating radiation ulcers than JAKi, and there is great potential to treat radiation ulcers by developing safe and effective drugs that inhibit SASP.

A major challenge in treating radiation ulcers is repairing the ulcerated mucosa without promoting cancer, as KGF was shown to promote growth of human epithelial tumor cells (45). The development of potential agents that mitigate radiation ulcers without accelerating tumor growth is intensively needed in oncological supportive care. A related report concluded that Smad7 prevents radiotherapy-induced oral mucositis but does not prompt tumor growth (46). Here, we showed that DQ treatment alleviated radiation-induced ulcers by selectively eliminating senescent cells. Moreover, DQ also enhanced radiosensitivity and reduced proliferative capacity in representative CAL27 and MCF-7 cells.

In summary, we demonstrated that senescent cells persist in radiation ulcers, and clearance of senescent cells by DQ can effectively mitigate this painful side effect. Moreover, DQ treatment can enhance cancer cell radiosensitivity. Our results indicate that elimination of senescent cells is a potential therapeutic method to mitigate radiation ulcers.

## Data Availability Statement

The raw data supporting the conclusions of this article will be made available by the authors, without undue reservation, to any qualified researcher.

## Ethics Statement

The animal study was reviewed and approved by the Ethics Committees of Army Medical University (AMU). The studies involving human participants were reviewed and approved by the ethics committee of Hunan Cancer Hospital. The patients/participants provided written informed consent for participation was required for this study in accordance with the national legislation and the institutional requirements.

## Author Contributions

CS, HW, and ZW designed, carried out, and analyzed data from most of the experiments and wrote the manuscript with input from all co-authors. CS conceived and supervised the study. YH, QJ, and ML performed experiments. YZ and XS collected clinical samples. YW and PL analyzed and interpreted data from experiments. All authors discussed the results and commented on the manuscript.

### Conflict of Interest

The authors declare that the research was conducted in the absence of any commercial or financial relationships that could be construed as a potential conflict of interest.
