# Supplementary material for: Senolytics (DQ) Mitigates Radiation Ulcers by Removing Senescent Cells
Source: Front Oncol. 2020 Feb 14;9:1576. doi: 10.3389/fonc.2019.01576 (PMC7034035; doi:10.3389/fonc.2019.01576)
Supplement: Supplementary file 1 [file Data_Sheet_1.PDF]

Supplementary figures:

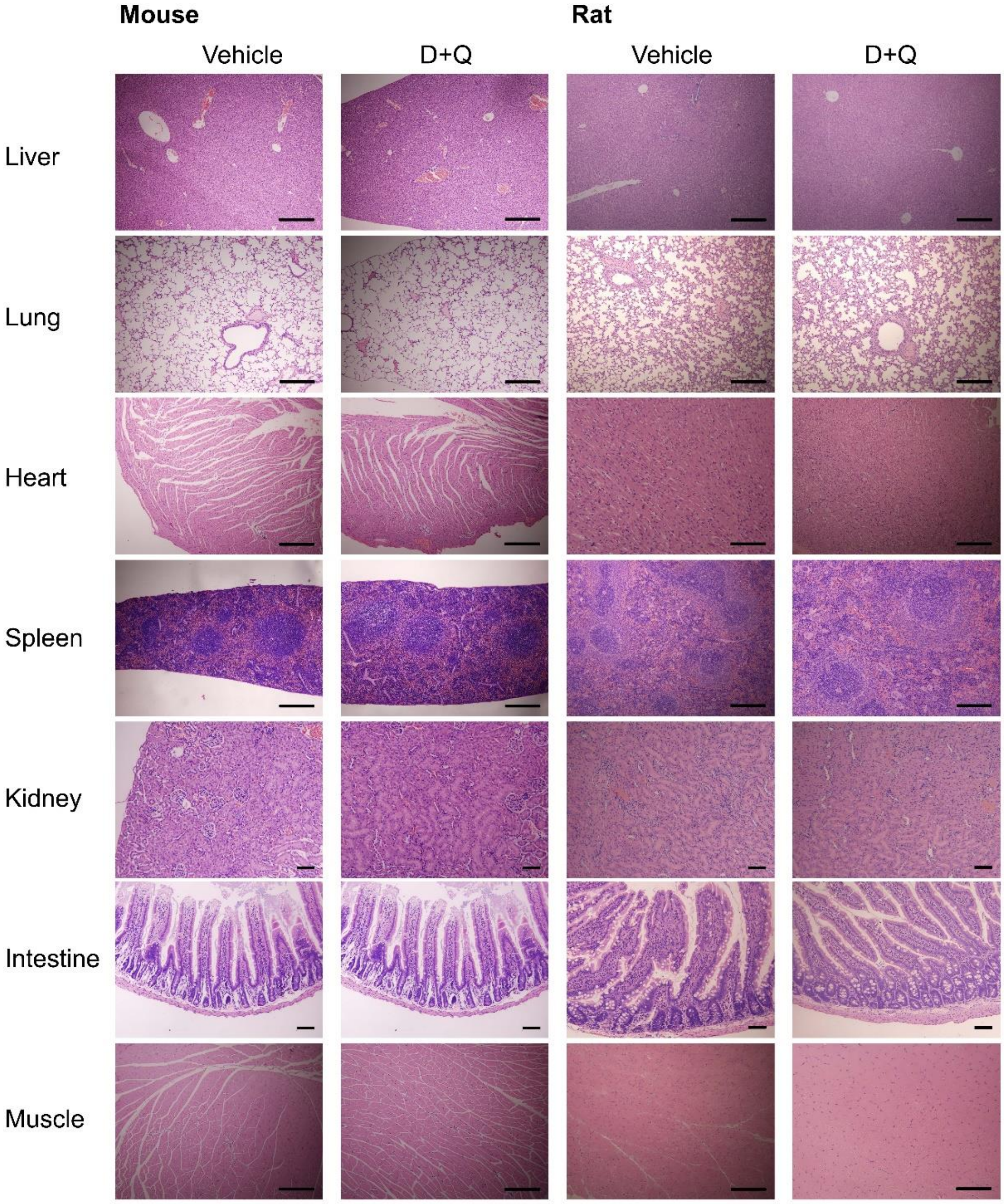

Supplementary figure 1. Mice/Rats were injected intraperitoneally with vehicle control or DQ every day for 5 days. Lung, liver, spleen, heart, kidney intestine and muscle were collected at day 15 for HE staining. Bars represent 100μm (liver, heart, spleen, lung, muscle), 50μm (kidney, intestine).

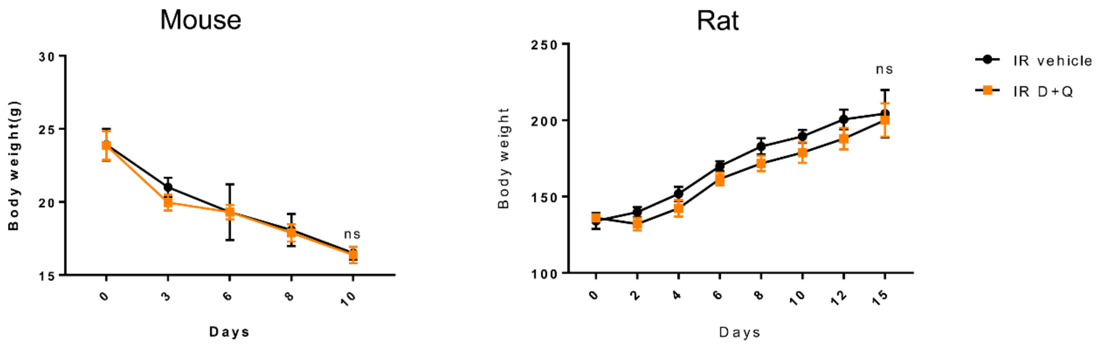

Supplementary figure 2. Body weight changes of mice and rats after radiation and DQ treatment.

Supplementary Table 1. Sequences of the primers used for qRT-PCR

Gene	Forward sequences	Reverse sequences
Human β-Actin	5-GAGAAAATCTGGCACCACACCT-3	5-GCACAGCCTGGATAGCAACGTA-3
Human PAI1	5-GCACCACAGACGCGATCTT-3	5-ACCTCTGAAAAGTCCACTTGC-3
Human MCP1	5-ATGAAAGTCTCTGCCGCCCT-3	5-CAGCAGGTGACTGGGGCATT-3
Human MMP3	5-AGTCTTCCAATCCTACTGTTGCT-3	5-TCCCCGTCACCTCCAATCC -3
Human MMP12	5-GGAATCCTAGCCCATGCTTTT-3	5-CATTACGGCCTTTGGATCACT-3
Human IL-1 α	5-AGATGCCTGAGATACCCAAAACC-3	5-CCAAGCACACCCAGTAGTCT-3
Human IL-1β	5-ATGATGGCTTATTACAGTGGCAA-3	5-GTCGGAGATTCGTAGCTGGA-3
Human IL-6	5-ACTCACCTCTTCAGAACGAATTG-3	5-CCATCTTTGGAAGGTTCAGGTTG-3
Human IL-8	5-ACTGAGAGTGATTGAGAGTGGAC-3	5-AACCCTCTGCACCCAGTTTTC -3
Human IL-10	5-GACTTTAAGGGTTACCTGGGTTG-3	5-TCACATGCGCCTTGATGTCTG -3
Human TNF- α	5-GAGGCCAAGCCCTGGTATG-3	5-CGGGCCGATTGATCTCAGC -3
Mouse β-Actin	5-CGTGCGTGACATCAAAGAGAAG-3	5-CAAGAAGGAAGGCTGGAAAAGA-3
Mouse PAI1	5-TCTGGGAAAGGGTTCACTTTACC-3	5-GACACGCCATAGGGAGAGAAG-3
Mouse MCP1	5-TAAAAACCTGGATCGGAACCAAA-3	5-GCATTAGCTTCAGATTTACGGGT-3
Mouse	5-GCATTAGCTTCAGATTTACGGGT-3	5-GGCCTGGAACAGTCTTGGC-3

MMP3		
Mouse MMP12	5-GGGCTGCTCCCATGAATGAC-3	5-CCAGAGTTGAGTTGTCCAGTTG-3
Mouse IL-1α	5-TCTATGATGCAAGCTATGGCTCA-3	5-CGGCTCTCCTTGAAGGTGA-3
Mouse IL-1β	5-GAAATGCCACCTTTTGACAGTG-3	5-TGGATGCTCTCATCAGGACAG-3
Mouse IL-6	5-CTGCAAGAGACTTCCATCCAG-3	5-AGTGGTATAGACAGGTCTGTTGG-3
Mouse IL-8	5-TCGAGACCATTTACTGCAACAG-3	5-CATTGCCGGTGGAAATTCCTT-3
Mouse IL-10	5-CTTACTGACTGGCATGAGGATCA-3	5-GCAGCTCTAGGAGCATGTGG-3
Mouse TNF-α	5-CAGGCGGTGCCTATGTCTC-3	5-CGATCACCCCGAAGTTCAGTAG-3
Rat β-Actin	5-CTAAGGCCAACCGTGAAAAGAT-3	5-ACCAGAGGCATACAGGGACAAC-3
Rat PAI1	5-CGCCTCCTCATCCTGCCTAA-3	5-ACGCCACTGTGCCGCTCT-3
Rat MCP1	5-TGCTGACCCCAATAAGGAATG-3	5-GCTTGAGGTGGTTGTGGAAAA-3
Rat MMP3	5-TGGACCCTGAGACCTTACCAAT-3	5-TTTTCGCCAAAAGTGCCTGT-3
Rat MMP12	5-GTAACTGGGCAACTGGACACCT-3	5-CATCTTGACCTCTGGGGCACT-3
Rat IL-1α	5-CGGAAACACCAAAACTCATCACA-3	5-GTAACTGGGCAACTGGACACCT-3
Rat IL-10	5-CACTGCTATGTTGCCTGCTCTTA-3	5-ATGTGGGTCTGGCTGACTGG-3
Mouse CDKN2A	5-CGAGAACGGTGGAACTTTGAC-3	5-CCAGGGCTCAGGTAGACCTT-3
Mouse CDKN1A	5-CGCAGGTTCTTGGTCACTGT-3	5-TGTTCACGAAAGCCAGAGCG-3

Abbreviations: CDKN2A, cyclin dependent kinase inhibitor 2A (aliases: p16); CDKN1A, cyclin dependent kinase inhibitor 1A (aliases: p21); PAI1, phosphoribosyl anthranilate isomerase 1; MCP1, mast cell proteinase-1; MMP3, matrix metalloproteinases 3; MMP12, matrix metalloproteinases 12; IL-1α, interleukin 1 alph; IL-1β, interleukin 1 beta; IL-6, interleukin 6; IL-8, interleukin 8; IL-10, interleukin 10; TNF-α, tumour necrosis factor alpha.
